# From famine to feast: a seesawing platelet count in severe iron deficiency anemia

**DOI:** 10.1007/s12308-025-00676-0

**Published:** 2025-12-09

**Authors:** Allison Weisnicht, Eduard Matkovic

**Affiliations:** 1 American Family Children’s Hospital, Department of Pediatrics, University of Wisconsin-Madison, Madison, WI USA; 2 School of Medicine and Public Health, Department of Pathology, University of Wisconsin-Madison, Madison, WI USA; 3 Department of Pathology and Laboratory Medicine, University of Wisconsin Hospital, D4/252 – MC2472, 600 Highland Ave, Madison, WI 53792-8550 USA

**Keywords:** Iron deficiency, Thrombocytopenia, Thrombocytosis

## Abstract

A 22-month-old previously healthy girl presented with profound microcytic anemia and severe thrombocytopenia due to iron deficiency. Following transfusions and iron supplementation, her platelet count rose dramatically, peaking at 3.5 million/µL before gradually normalizing over several months. This case highlights the rare presentation of iron deficiency with severe thrombocytopenia, which can mimic bone marrow disorders. It also illustrates the phenomenon of rebound thrombocytosis after iron repletion, emphasizing the need for close platelet monitoring to mitigate potential thrombotic risk.

A previously healthy 22-month-old girl presented with severe microcytic anemia and thrombocytopenia (hemoglobin 2.1gm/dL, MCV 59 fL, platelets 9 × 10^3^ cells/µL) (Fig. 1A). Iron deficiency was determined as the cause of bi-cytopenia. She received red blood cell and platelet transfusions and was started on oral iron supplementation. A follow-up CBC 1 week later showed improved hemoglobin and marked thrombocytosis with a platelet count of 3.5 million per microliter (Fig. 1B). She underwent further evaluations including a bone marrow biopsy. Bone marrow was normocellular with trilineage hematopoiesis, 2% blasts, abundant megakaryocytes, and absent storage iron (Fig. 1C and D). Five weeks later, her platelet count fell back to 264 K/µL. Her clinical course was prolonged with oscillating platelet counts and ferritin levels following supplementation with IV ferric carboxymaltose infusions (Fig. 2). Eventual normalization of her CBC and stabilization of platelet count occurred several months after presentation.

**Fig. 1:**
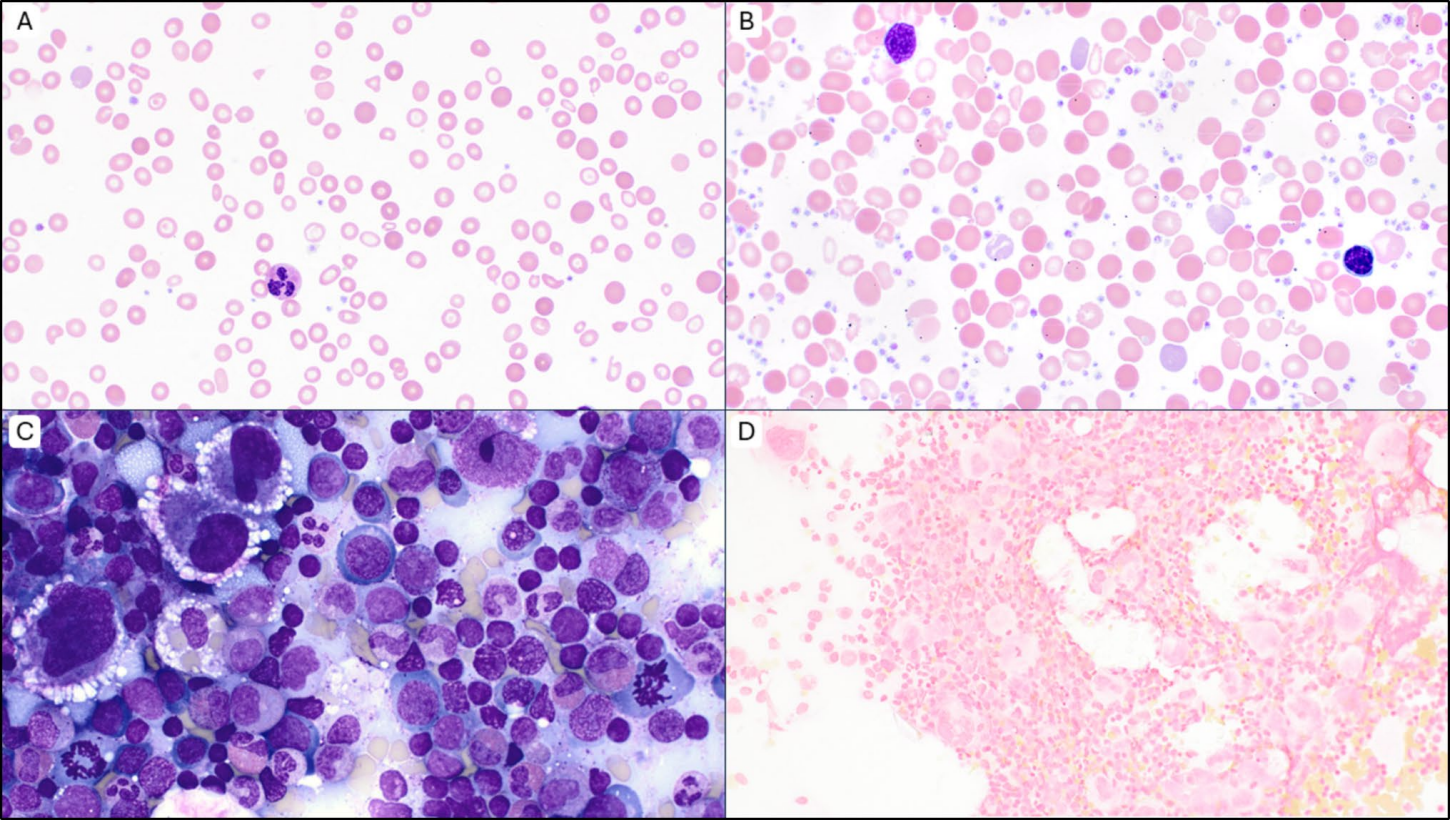
Peripheral blood smear shows features of iron deficiency anemia and thrombocytopenia (Wright-Giemsa stain, 100× objective) (**A**). Marked thrombocytosis develops following iron supplementation (Wright-Giemsa-stained peripheral blood smear, 100× objective) (**B**). Normal cellular bone marrow aspirate; Giemsa-stained smear examined at 50× objective (**C**). Bone marrow with decreased iron stores (Prussian blue stain, 50× objective) (**D**)

**Fig. 2:**
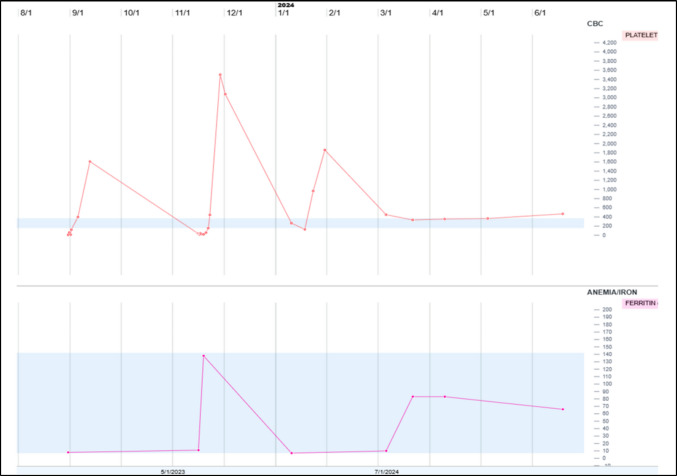
Graph of platelet count trend (top) in relation to ferritin levels (bottom) corresponding to iron repletion status

Severe thrombocytopenia associated with iron deficiency is a rare phenomenon, particularly in pediatric populations. Although iron deficiency anemia is most commonly associated with thrombocytosis, a subset of patients—especially those with severe iron deficiency—may develop severe thrombocytopenia [1–3]. This can mimic more serious bone marrow pathologies, but the clinical course is distinct. This case also showcases a remarkable rebound thrombocytosis, with transient platelet overshoot phenomenon that can occur after iron repletion. The mechanism is not fully understood, but the phenomenon underscores the importance of monitoring platelet counts with iron repletion, specifically in patients at risk for thrombotic complications.

## Data Availability

No datasets were generated or analyzed during the current study.
